# Accumulation of Polyphenols and Associated Gene Expression in Hairy Roots of *Salvia viridis* Exposed to Methyl Jasmonate

**DOI:** 10.3390/ijms25020764

**Published:** 2024-01-07

**Authors:** Izabela Grzegorczyk-Karolak, Marta Krzemińska, Renata Grąbkowska, Jan Gomulski, Cezary Żekanowski, Katarzyna Gaweda-Walerych

**Affiliations:** 1 Department of Biology and Pharmaceutical Botany, University of Lodz, 90-151 Lodz, Poland; marta.wojciechowska2@stud.umed.lodz.pl (M.K.); renata.grabkowska@umed.lodz.pl (R.G.); jan.gomulski@umed.lodz.pl (J.G.); 2 Department of Neurogenetics and Functional Genomics, Mossakowski Medical Research Institute, Polish Academy of Sciences, 02-106 Warsaw, Poland; c.zekanowski@imdik.pan.pl (C.Ż.); kgaweda@imdik.pan.pl (K.G.-W.)

**Keywords:** elicitation, hairy roots, HPLC, organ culture, phenylpropanoid pathway, rosmarinic acid, real-time PCR (RT-PCR) analysis, gene expression

## Abstract

Methyl jasmonate (MJA), a signaling molecule in stress pathways, can be used to induce secondary metabolite synthesis in plants. The present study examines its effects on the growth of *Salvia viridis* hairy roots, and the accumulation of bioactive compounds, and correlates it with the expression of genes involved in the phenylpropanoid pathway. To our knowledge, this study represents the first exploration of elicitation in *S. viridis* culture and the first comprehensive analysis of MJA’s influence on such a wide array of genes within the polyphenol metabolic pathway in the *Salvia* genus. Plants were treated with 50 and 100 µM MJA, and samples were collected at intervals of one, three, five, and seven days post-elicitation. HPLC analysis revealed that MJA stimulated the accumulation of all tested compounds, with a 30% increase (38.65 mg/g dry weight) in total polyphenol content (TPC) on day five. Quantitative real-time polymerase chain reaction (RT-PCR) analysis demonstrated a significant increase in the expression of the phenylpropanoid pathway genes—*TAT* (tyrosine aminotransferase), *HPPR* (4-hydroxyphenylpyruvate reductase), *PAL* (phenylalanine ammonia-lyase), *C4H* (cinnamic acid 4-hydroxylase), *4CL* (4-coumarate-CoA ligase), and *RAS* (rosmarinic acid synthase)—following MJA treatment. For the majority of the genes, this increase was observed after the first day of treatment. Importantly, our present results confirm strong correlations of the analyzed gene expression with polyphenol biosynthesis. These findings support the notion that hairy roots provide a promising biotechnological framework for augmenting polyphenol production. Additionally, the combination of elicitor treatment and transgenic technology emerges as a viable strategy to enhance the biosynthesis of these valuable metabolites.

## 1. Introduction

Jasmonic acid (JA) and its derivatives, including the methyl ester (MJA), are signaling molecules formed from α-linolenic acid in different branches of the lipoxygenase pathway. They regulate many aspects of plant growth and development, such as seed germination, root and whole plant growth, stamen development, flowering, and senescence [1,2]. In addition, jasmonates activate the defense mechanisms of plants in response to various pathogens and environmental stressors, such as water deficit, low temperature, nutrient deficiency, and salinity. Exogenously applied JA and its methyl ester, MJA, are capable of triggering transcriptional reprogramming. In a wide range of plant species, they coordinate the activation of genes associated with the formation of secondary metabolites that allow cells to cope with such stresses [3,4]. Some studies suggest that mitigating the negative impact of various types of stress factors using MJA allows plants to increase their tolerance to unfavorable environmental conditions [5].

Many studies have examined the effect of MJA on various metabolic pathways in a range of plant species. It has also been shown to induce the production of antioxidant defense enzymes and secondary metabolites in plant organ and cell cultures. For example, MJA has demonstrated a positive effect on ginsenoside production in ginseng cell suspension and hairy root cultures [6], glycyrrhizin production in *Glycyrrhiza inflata* hairy root culture [7], and tropane alkaloids in transgenic *Hyoscyamus niger* hairy root culture [8].

Numerous studies have also found this elicitor to increase the accumulation of polyphenols, including rosmarinic acid (RA), in plants. Due to the great importance of RA [9,10], there has been great interest in better understanding its production both in vivo and in vitro. Successful commercial production depends on the metabolic response to the type and concentration of the elicitor, and the exposure time. It has been shown that MJA application increases the accumulation of RA in *Lithospermum erythrorhizon* cell suspension [11]. Elicitation with MJA enhanced RA production 2.4-fold in *Lavandula* culture compared to non-elicited cells [12], and 3.4-fold in *Coleus forskohlii* hairy roots compared to controls [13].

Sage species can also accumulate RA upon MJA treatment, providing an excellent model to investigate polyphenol biosynthesis and the regulation of genes within this metabolic pathway. Methyl jasmonate treatment has been reported to stimulate the biosynthesis of caffeic acid (CA) and RA in the hairy roots of *Salvia przewalskii* [14], and the regenerated shoots of *Salvia virgata* accumulated 70% more RA on day 3 after exposure to MJA compared to a control group [15]. Although studies have confirmed the effect of MJA on RA biosynthesis in several sage species, the transcription levels of genes involved in RA biosynthesis have not been extensively studied. A few reports have shown that the exogenous application of MJA induced the expression of genes associated with polyphenol biosynthetic pathways; for example, it has been found that increased *PAL* expression induced by MJA preceded an increase in RA accumulation [11,16,17].

Different *Salvia* species may exhibit diverse expression patterns of genes in the phenylpropanoid pathway, leading to variations in the production of secondary metabolites like flavonoids, lignins, and phenolic acids. For this reason, this study examines the effects of MJA on the biosynthesis of RA and other polyphenols in *Salvia viridis* hairy root culture. *S. viridis* is an annual herb which grows in the Mediterranean area. Its aerial parts have been used in Turkish medicine for their anti-inflammatory and antiseptic properties [18]. Among its secondary metabolites, phenolic acids, phenylpropanoids, and terpenoids are believed to predominate [19], and *S. viridis* hairy roots have been found to be a promising potential source of phenolic acids [20]. In this study, we also evaluate the transcriptional expression profiles of six genes involved in polyphenol accumulation in the presence of MJA. We correlate these profiles with the production of individual compounds, aiming to further elucidate the key mechanisms within the polyphenol synthesis pathway, which are currently understood only at a basic level. The identification of key regulatory genes across *Salvia* sp. can provide targets for genetic engineering interventions aiming to boost the production of metabolites. To our knowledge, this is the first study to employ elicitation in *S. viridis* culture and the first one to analyze the impact of MJA on such a wide range of genes controlling the polyphenol metabolic pathway in a member of the *Salvia* genus.

## 2. Results and Discussion

### 2.1. Effect of MJA on Biomass Accumulation and Phenolic Content in Salvia viridis Hairy Roots

**Figure 1:**
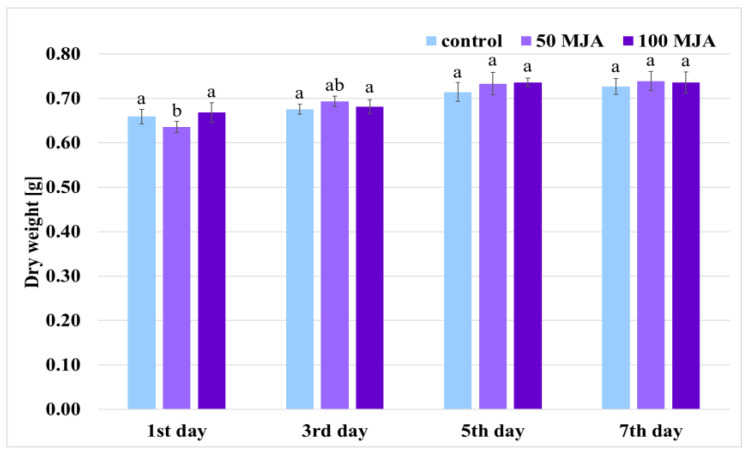
Growth of *Salvia viridis* culture 1, 3, 5, and 7 days after treatment with 50 μM and 100 μM MJA. Transformed roots treated with only 10 μL of ethanol were used as controls; the values are given as mean ± standard error of three independent experiments. If at least one letter is the same between two treatments, the difference is not statistically significant (*p* < 0.05); the results were obtained via one-way ANOVA analysis, followed by a post hoc Tukey’s test.

Two concentrations of methyl jasmonate were selected for application: 50 and 100 μM. These values have been highly effective in previous studies [12,21,22]. The elicitor was added on day 29 of passage, in the plateau phase; this day was established according to previous studies [23]. The dry weight (DW) of culture was measured every two days from day 1 to 7 after introducing the elicitor to the growth medium. The addition of MJA was found to generally have no negative effect on culture growth (Figure 1). The roots continued to grow over the next seven days, ultimately reaching from 0.727 to 0.739 g DW (depending on the treatment) on the 36th day of cultivation. No differences in culture growth were observed between treatments, apart from root dry weight on the first day after supplementation with 50 µM MJA (Figure 1).

Many studies have reported the inhibition of plant growth under the influence of elicitors; indeed, experiments on *Taxus* media and *Lavandula* cultures indicate the inhibition of cell growth under MJA treatment [12,24], and *Raphanus sativus* growth was slowed during MJA treatment compared to untreated controls [25]. In addition, the fresh weight of *Salvia castanea* hairy root culture was lower in the presence of exogenous MJA [26]. This inhibitory effect most likely occurs as a result of signaling from MJA, informing the plant to focus on defense against stress factors at the expense of growth, thus increasing the chances of survival in the natural environment [2]. As such, it is necessary to determine the appropriate moment of MJA supplementation so as not to significantly interfere with culture growth.

**Figure 2:**
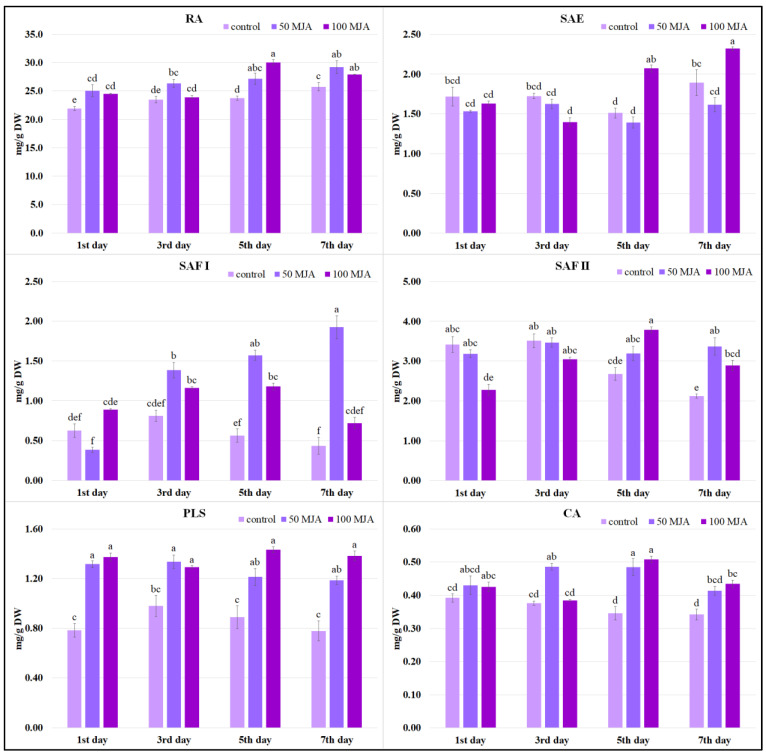
Polyphenol content obtained in *Salvia viridis* hairy roots 1, 3, 5 and 7 days after elicitation with 50 μM and 100 μM of MJA. Transformed roots treated with only 10 μL of ethanol were used as controls; the values are given as mean ± standard error of three independent experiments. If at least one letter is the same between two treatments for the same compound, the difference is not statistically significant (*p* < 0.05); the results were obtained via one-way ANOVA analysis, followed by a post hoc Tukey’s test. CA—caffeic acid; PLS—prolithospermic acid; SAE—salvianolic acid E; RA—rosmarinic acid; SAF I and II—salvianolic acid F isomers I and II.

To assess the effects of MJA elicitation on polyphenol biosynthesis, their contents in the hairy root samples were measured via HPLC analysis one, three, five, and seven days after elicitation. Six phenolic acids were tested: rosmarinic acid, salvianolic acid E (SAE), isomers of salvianolic acid F I and II (SAF I and SAF II), prolithospermic acid (PLS), and caffeic acid (CA), as well as total phenolic content. It was found that MJA treatment had a beneficial effect on secondary metabolite production in the hairy roots of *Salvia viridis* (Figure 2). However, the influence on individual compounds depended on elicitation time and elicitor concentration.

The level of PLS in the roots increased two-fold compared to the controls after the first day of elicitation. This level was maintained for up to seven days after the elicitation of MJA.

The production of RA, the dominant compound in the phytochemical profile of *S. viridis* hairy roots (Figure 2), was also stimulated from the first day after MJA treatment. However, its level peaked five to seven days after elicitation, with the highest value (30.1 mg/g DW) occurring on day five with 100 μM MJA, i.e., 27% higher than the control.

For most metabolites, production was most strongly stimulated five days after MJA treatment. At this point, all polyphenol levels were higher than in the non-MJA-treated culture, regardless of the elicitor concentration. Salvianolic acid E was the only metabolite for which a concentration of 50 μM MJA was insufficient to increase biosynthesis (Figure 2); however, 100 μM MJA treatment yielded a significant increase in SAE level from day five, reaching a level of 2.32 mg/g DW in the roots on day seven.

In contrast, optimal biosynthesis of the SAF I isomer was obtained at the lower (50 μM) MJA concentration (Figure 2). In the 50 μM MJA-treated culture, the content of SAF I isomer after seven days was almost three times higher than that in the 100 μM MJA treatment and four times higher than in the control.

**Figure 3:**
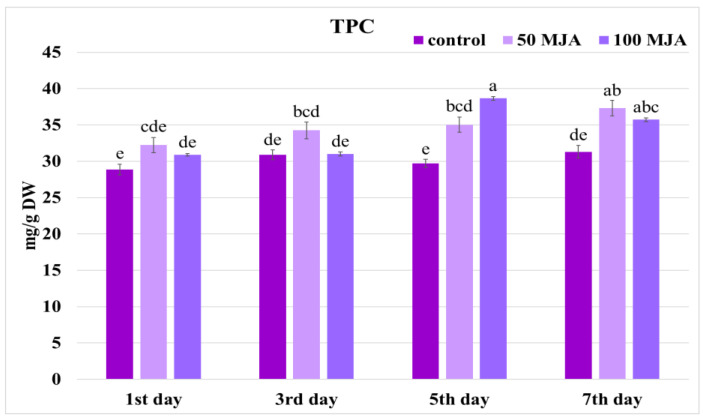
Total phenolic content obtained in *Salvia viridis* hairy roots 1, 3, 5, and 7 days after elicitation with 50 μM and 100 μM of MJA. Transformed roots treated with only 10 μL of ethanol were used as controls; the values are given as mean ± standard error of three independent experiments. If at least one letter is the same between two treatments, the difference is not statistically significant (*p* < 0.05); the results were obtained via one-way ANOVA analysis, followed by a post hoc Tukey’s test.

Finally, the highest total phenol content (TPC) (38.65 mg/g DW) was obtained after five days of elicitation with 100 μM of MJA; the value was 30% greater than in the control hairy roots (Figure 3). A similar level of phenolic compounds was observed seven days after supplementation, regardless of the elicitor concentration used.

Stimulators such as MJA have been found to increase RA accumulation in *Lamiaceae* cell cultures, such as those of *Lavandula vera* [12], *Lithospermum erythrorhizon* [11,27], and *Coleus blumei* [16]. Some studies have also examined its effect on plant organs, including transformed roots. Treatment with 10 μM MJA increased RA accumulation in *S. miltiorrhiza* hairy roots 1.36-fold [28]. A total of 50 μM MJA induced 1.60-fold and 2.2-fold increases in RA accumulation in *Salvia verticillata* and *Salvia officinalis* leaves, respectively [29]. Both 50 and 100 μM MJA increased RA production in a shoot culture of *S. officinalis*, with an optimal elicitation time of five days [22]. Elsewhere, 400 μM MJA promoted 1.3-fold RA accumulation in *S. przewalskii* hairy roots, with the content peaking on the third day after treatment [14]. Whereas 100 and 500 μM MJA increased the RA content in *Coleus forskohlii* hairy roots [13].

These data, along with our current findings, indicate that methyl jasmonate is an effective elicitor for RA and other polyphenols in various in vitro plant cultures, including the newly tested *Salvia viridis*. However, the optimal elicitor concentration and time to peak metabolite accumulation vary according to the species.

### 2.2. Gene Expression Analysis

**Figure 4:**
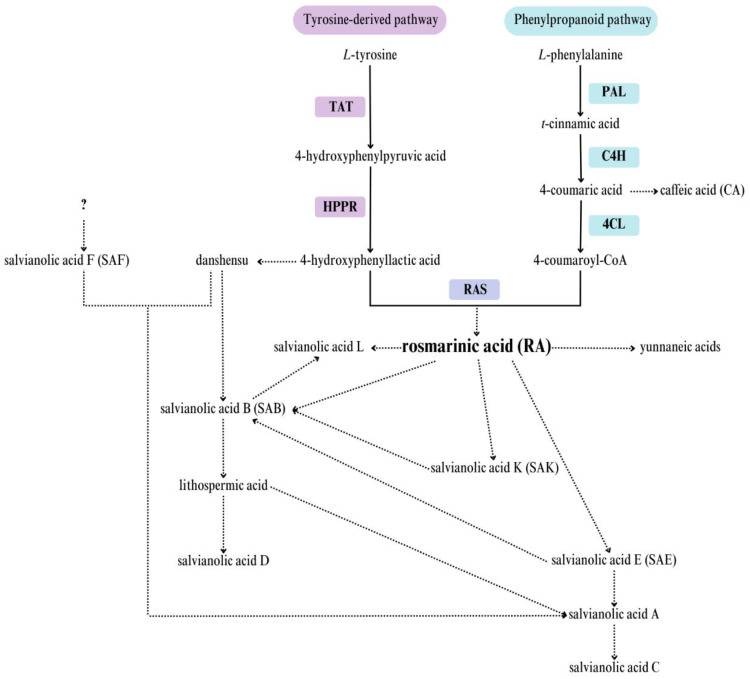
The biosynthetic pathway of phenolic acids in plants. TAT: tyrosine aminotransferase; HPPR: 4-hydroxyphenylpyruvate reductase; PAL: phenylalanine ammonia-lyase; C4H: cinnamic acid 4-hydroxylase; 4CL: 4-coumarate-CoA ligase; RAS: rosmarinic acid synthase [28,30,31].

Phenolic acids are important bioactive compounds in *Salvia* species, and their biosynthetic regulation has been the subject of investigation in several studies. [28,30,31]. Their biosynthesis starts with tyrosine and phenylalanine, and the pathway consists of two parallel branches involving five enzymes: tyrosine aminotransferase (TAT), 4-hydroxyphenylpyruvate reductase (HPPR), phenylalanine ammonia-lyase (PAL), cinnamic acid 4-hydroxylase (C4H), and 4-coumarate-CoA ligase (4CL) (Figure 4). The two pathways later converge through the reaction of the two resulting compounds, viz., coumaroyl-CoA and 4-hydroxyphenyllactic acid, catalyzed by rosmarinic acid synthase (RAS) to produce RA. Most of the genes encoding these enzymes have been reported to be responsive to different elicitors, such as MJA, salicylic acid, yeast, metal ions, and UVB radiation, indicating that these genes may be regulated by a similar set of transcription factors participating in these signaling pathways [14,17,29,32,33,34]. MJA-driven changes in gene expression in this pathway may influence the accumulation of polyphenols.

Real-time PCR (RT-PCR) was used to analyze the expression of the six genes (*TAT*, *RAS*, *C4H*, *4CL*, *HPPR*, *PAL*) involved in different steps of the phenolic acid biosynthetic pathway (Figure 4). Since a previous study showed that the level of actin remained stable in different tissues and organs of *S. miltiorhhiza* and other *Salvia* species [14,35], it was used as a reference gene to normalize gene expression in our study.

**Figure 5:**
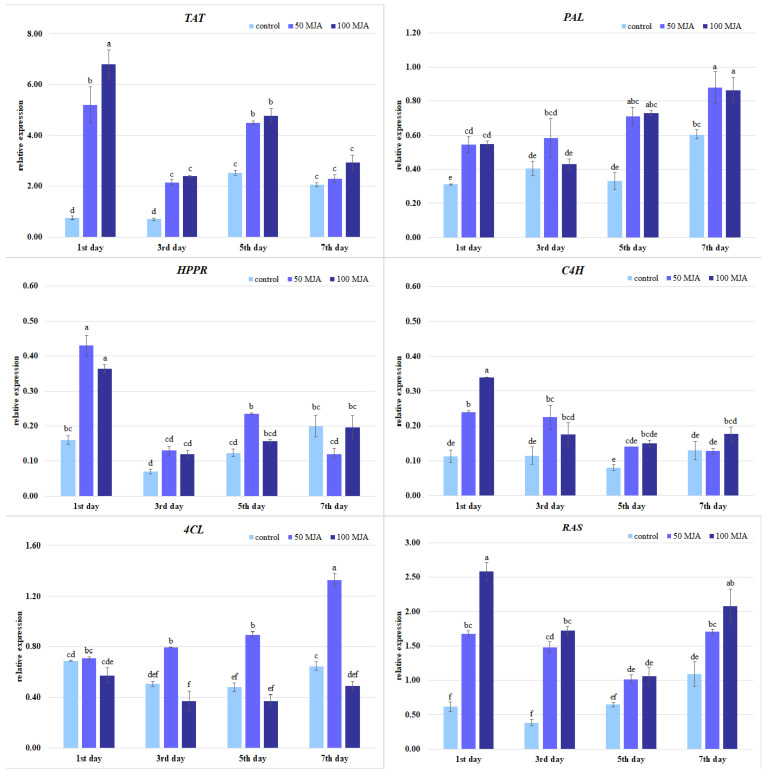
The expression levels of polyphenol biosynthetic genes in *Salvia viridis* hairy roots 1, 3, 5, and 7 days after elicitation with 50 μM and 100 μM of MJA. Transformed roots treated with 10 μL of ethanol were used as controls; the values are given as mean ± standard error of three independent experiments. If at least one letter is the same between two treatments for the same gene, the difference is not statistically significant (*p* < 0.05); the results were obtained via one-way ANOVA analysis, followed by a post hoc Tukey’s test. *TAT*: tyrosine aminotransferase, *HPPR*: 4-hydroxyphenylpyruvate reductase, *PAL*: phenylalanine ammonia-lyase, *C4H*: cinnamic acid 4-hydroxylase, *4CL*: 4-coumarate-CoA ligase, *RAS*: rosmarinic acid synthase.

It was found that both elicitor concentration and exposure time influenced the level of gene expression in *S. viridis* culture (Figure 5). After one day, higher transcript levels were observed for all tested genes, except for *4CL*, in MJA-treated hairy roots compared to the untreated controls. The greatest increase in gene expression was noted for *TAT*, which exhibited a nine-fold increase following treatment with 100 μM MJA, and a seven-fold increase after treatment with 50 μM MJA, compared to the control cultures (Figure 5).

Likewise, *HPPR* expression substantially increased after the first day of treatment with 50 µM (2.7-fold) and 100 µM MJA (2.3-fold). Both MJA concentrations were also effective in stimulating the expression of *CH4*, with a 2–3-fold increase in expression on the first day followed by gradual normalization over the following days. In contrast, the expression of *PAL*, the initial enzyme of the phenylalanine pathway, showed a slow but consistent increase over time, peaking on day five. At this point, its expression was almost twice as high as that of the control. Interestingly, *4CL* demonstrated increased relative expression only at the lower elicitor concentration (from days three to seven). In the case of *RAS*, a downstream pathway gene, the greatest increase in expression was observed on day one of elicitation, when MJA treatments reached 3.9- to 4.5-fold higher levels than the controls, depending on the elicitor concentration. Elevated expression (1.6–1.9-fold) persisted throughout the following days of the experiment.

TAT is a key enzyme at the beginning of the RA biosynthetic pathway (Figure 4). Alongside HPPR, it constitutes a branch of the tyrosine-derived pathway. The expression of *TAT* after the MJA treatment of *S. viridis* followed a similar trend to previous observations for *S. miltiorrhiza* leaves, with significantly increased transcript levels observed between 4 and 72 h after treatment [36]. Similarly, Yan et al. [33] observed TAT upregulation in YE- and Ag-treated cultures of *S. miltiorrhiza* hairy roots after eight hours, with a peak at 12 h of elicitor treatment; the enzyme activity then declined slightly, but remained higher than the control values for up to four days. In the elicitor-treated culture, TAT activity peaked at a value about 1.5 times higher than the control values [33]. In *Anchusa officinalis* cell suspension cultures, TAT activity was found to correlate positively with the level of RA biosynthesis during the linear growth phase of the culture cycle [37].

Moreover, *TAT* expression was correlated positively with TPC accumulation for *S. officinalis* [29] and with TPC and RA production (*r* = 0.96 and *r* = 0.87, respectively) in *Melissa officinalis* shoot cultures [38]. These data are consistent with our present results, where *TAT* expression strongly correlated with TPC levels, including RA and some other polyphenols (Table 1). The increase in *TAT* expression on the first day after treatment was most strongly associated with the accumulation of TPC (0.9876), RA (0.9767), PLS (0.9875), and SAF II (0.9529) on the fifth day. A similar delay was observed for the majority of the other polyphenols and could be attributed to the fact that product synthesis takes time. Likewise, an increase in *TAT* and *PAL* expression was found to precede peak RA accumulation by around four days in *Orthosiphon aristatus* cell suspension cultures treated with yeast extract as an elicitor [39]. All these findings point to *TAT* as an important gene in phenolic acid production in plants.

Similarly, MJA treatments were found to upregulate the transcription level of *HPPR*, the second gene of the tyrosine pathway, in the hairy roots of *Salvia przewalski* on day 3 after treatment, i.e., a little later than in the present study [14]. In *Lithospermum erythrorhizon* cell suspension cultures activated by MJA, a strong transient increase was observed in *HPPR* and *PAL* activity in the MJA-treated cells, which correlated closely with RA accumulation, whereas *TAT* activity exhibited only a slight increase [11]. Fatemi et al. [17] also reported MJA-dependent stimulation of *TAT*, *PAL*, *HPPR*, and *RAS* gene expression in nodal segment cultures of *Satureja khuzistanica*. Our present results further confirm very strong correlations of the expression of these four genes and *C4H* with polyphenol biosynthesis (Table 1). As in the case of *TAT*, *C4H* and *RAS* demonstrated the strongest correlations with total phenol and RA accumulation on day five and for *HPPR* on day seven.

It has previously been found that the expression of genes in the upstream part of the pathway (*PAL*, *C4H*, *4CL*, *TAT*, and *HPPR*) in hairy roots of *S. miltiorrhiza* was promoted by Ag ions; they reached their maximum on the first day after treatment, while RA content was significantly increased on day 6 after treatment (1.3 times those in the control) [40]. Elsewhere, UVB radiation enhanced the expression of *PAL*, *TAT*, and *RAS*, with the highest level in leaves of *S. verticillata* on the 10th day of exposure, and stimulated the accumulation of phenolic acids such as RA and CA [34]. Moreover, a previous study on *Melissa* suspension culture found that *4CL* expression correlated with RA content, with gene expression peaking just before elevated production [32]. In the present study, although both 50 μM and 100 μM MJA increased total phenolic content (Figure 3), only lower MJA concentration stimulated *4CL* expression, which could suggest that this gene is highly sensitive to regulation by MJA (Figure 5). The low correlation between *4CL* expression and polyphenol accumulation in *S. viridis* roots (Table 1) may also imply that the conversion of 4-coumaric acid to 4-coumaroyl-CoA catalyzed by 4CL is not a limiting step in elicitor-induced RA biosynthesis, and the non-elicited gene activity was already sufficient to catalyze the metabolic reaction.

In the present study, the expression of the polyphenolic pathway genes showed weak or no correlation with SAE levels in *S. viridis* culture; in some cases, a strong negative correlation was observed (Table 1). This may be caused by a bottleneck in the part of the pathway from RA to SAE, or the negative coupling of some genes with those of the final SAE transformations. Although this compound is believed to be formed through RA dimerization, the exact course of the reaction and the participating enzymes remain unknown (Figure 4). Therefore, our current findings provide only a preliminary insight into the regulation of RA biosynthesis by exogenous factors; further reactions involving conversion to other high-molecular-weight polyphenolic acids, as indicated in Figure 4, are currently unclear. The biosynthesis of an isomer of SAE—salvianolic acid B, the most abundant and bioactive form of the salvianolic acids—also remains unknown. Some reports suggest that it is a product of RA dimerization, while others indicate that it originates from the side branch of 4-hydroxyphenyllactic acid via an intermediate compound called danshensu [41].

Because our understanding of additional polyphenol metabolism pathways and the associated enzymes, as well as the influence of regulatory genes and potential feedback loops among genes, is limited, further extensive interdisciplinary investigation is required to establish the connections between all the factors affecting biosynthetic pathways.

## 3. Materials and Methods

### 3.1. Plant Material

Hairy roots of *Salvia viridis* were obtained using *Rhizobium rhizogenes* strain A4 as described by Grzegorczyk-Karolak et al. [20]. The K3 clone was selected for this study on the basis of its growth rate and polyphenolic content. Hairy roots were cultivated in optimized conditions, in 80 mL of WP [42] liquid medium, in Erlenmeyer flasks, on a rotary shaker at 70 rpm and 26 ± 2 °C, in the dark. Subcultures were carried out every 35 days.

### 3.2. Elicitor Preparation and Treatment

Methyl jasmonate (MJA) was dissolved in 96% ethanol. Elicitor from stock solutions was added (10 μL per flask) on day 29 of subculture using a sterile syringe filter (0.22 μm) to reach a final concentration of 50 or 100 μM. The optimal moment of elicitation was established based on the growth curve, i.e., when the stationary phase was reached [23]. The controls were non-elicited hairy roots treated with 10 μL of 96% ethanol. Both treatments were maintained on a rotary shaker at 70 rpm and 26 ± 2 °C, in the darkness. Three flasks were used for each treatment and the experiment was repeated three times (passage 62–64).

The hairy roots were harvested one, three, five, and seven days after elicitation, and their growth, expressed as dry weight (DW), and polyphenol accumulation were evaluated. To determine DW, the culture was drained of the liquid medium, and the plant material was frozen and lyophilized to a constant weight.

### 3.3. Preparation of Extracts for Phytochemical Analysis

Samples of lyophilized and micronized plant material (100 mg) were extracted with a 30 mL of methanol/water solution (4:1, *v*/*v*) in a sonication bath (Techpan, Warsaw, Poland) at 40° for 15 min. The extraction procedure was repeated twice with 15 mL of the same solvent. The combined extracts were evaporated to dryness under reduced pressure and stored at 4 °C until further analysis.

### 3.4. Quantitative Analysis

The phenolic acid content was determined by HPLC. The dry extracts were dissolved in 2 mL of methanol/water solution (4:1, *v*/*v*). The analysis itself was performed on Agilent Technologies 1290 Infinity HPLC apparatus (Santa Clara, CA, USA) with diode array detection (DAD) and an Eclipse XDB-C18 column (4.6 × 150 mm, 5 μm). The temperature was maintained at 35 °C, with a flow rate of 1.6 mL/min and an injection volume of 10 μL. The mobile phase consisted of acetonitrile as solvent A and water with 0.1% formic acid as solvent B. The elution profile was as follows: 0–5 min, 10% A and 90% B; 5–20 min, 18% A and 82% B; 20–25 min, 38% A and 62% B; 25–30 min, 100% A (isocratic elution). Compounds were detected at λ = 325 nm.

Authentic standards of caffeic acid (CA), rosmarinic acid (RA), and salvianolic acid B (SAB) (ChemFaces, Wuhan, China) were used for calibration. The following regression equations were obtained: for CA, y = 30.3230x − 20.3788 (R^2^ = 0.9639); for RA, y = 12.1228x + 1281.4666 (R^2^ = 0.9813); for SAB, y = 3.5352x − 3.3512 (R^2^ = 0.9957). For the phenolic acids lacking a standard, quantification was based on the calibration curve of a similar compound: SAB for PLS, SAE, SAF I, and SAF II. The amounts of the analyzed compounds were expressed as mg/g DW.

### 3.5. RNA Extraction and RT-PCR Analysis

RNA was extracted from fresh plant material harvested one, three, five, and seven days after elicitation according to the standard protocol for Total RNA Midi Kit (A&A Biotechnology, Gdańsk, Poland). RNA concentration was measured using a NanoDrop spectrophotometer (NanoReady Touch, Life Real Biotechnology Co., Ltd., Hangzhou, China). cDNA was synthesized from 1800 ng of total RNA with an NG dART RT cDNA synthesis kit (EURx Molecular Biology Products, Gdańsk, Poland) using random hexamer primers.

Quantitative real-time PCR analysis was performed with RT HS-PCR Mix SYBR (A&A BIOTECHNOLOGY, Gdańsk, Poland) (primers listed in Table 2) using the StepOne Plus system (Applied Biosystems, Foster City, CA, USA). Changes in gene expression were determined via the ∆∆Ct method using actin levels for normalization. The reaction used primers for the key genes known to encode the enzymes involved in phenolic acid biosynthesis, viz., phenylalanine ammonia-lyase (*PAL*), cinnamic acid 4-hydroxylase (*C4H*), hydroxycinnamate coenzyme A ligase (*4CL*), tyrosine aminotransferase (*TAT*), 4-hydroxyphenylpyruvate reductase (*HPPR*), and rosmarinic acid synthase (*RAS*). The six genes for the analysis were chosen based on published studies reporting other closely related plant species, since they encode known key enzymes controlling the major branches of the phenylpropanoid biosynthesis pathway [14,40,43]. The primers were synthesized at the Institute of Biochemistry and Biophysics of the Polish Academy of Sciences (Warsaw, Poland, https://oligo.ibb.waw.pl/, accessed on 15 July 2022). The results represent the mean of three biological replicates.

### 3.6. Statistical Analysis

All of the experiments were performed in triplicate and the results were calculated as the mean ± standard error (SE). The results of the treatments were compared using ANOVA, followed by Tukey’s post hoc test (Statistica13.1Pl, StatSoft, Krakow, Poland). The results were considered significant below *p* = 0.05.

## 4. Conclusions

*S. viridis* hairy roots may constitute a promising source for the production of RA and other polyphenols, which may be stimulated through supplementation with different MJA concentrations. The approach employed in this study was not only effective in obtaining higher polyphenol levels in culture, but our data also provided insight into the relationship between MJA-dependent polyphenolic acid accumulation and gene expression. Five days after elicitation with 100 µM MJA, the biosynthesis of RA and TPC was enhanced by around 30% compared to the controls, with no effect on culture growth. The observed changes in the expression of polyphenol biosynthesis-related genes in MJA-treated hairy root culture were both dose- and time-dependent. The upregulation of most genes in this metabolic pathway strongly correlated with an increase in the biosynthesis of the majority of polyphenols, including RA. Gene upregulation was often followed by an increase in polyphenolic accumulation after several days. Our findings provide an indication of how secondary metabolism in *S. viridis* responds to elicitors, and may help to develop more precise strategies for remodeling polyphenol pathways to further increase RA production in sage culture. However, such advances will require more in-depth knowledge of the regulatory mechanisms, which involve a wide range of factors nested in a complex plant signal transduction network. As such, further research should aim to comprehensively analyze the relationships between the different factors in metabolic pathways.

## Figures and Tables

**Table 1:** Correlation coefficients between gene expression and phenolic compounds at subsequent control moments (1, 3, 5, and 7 days after elicitation). Blue—strong (*r* > 0.7) or very strong (bold) correlation (*r* > 0.9); red—very strong negative feedback (*r* < −0.9).

		*TAT*				*PAL*				*HPPR*				*C4H*				*4CL*				*RAS*			
TPC		1	3	5	7	1	3	5	7	1	3	5	7	1	3	5	7	1	3	5	7	1	3	5	7
	1	0.7856	0.4091	**0.9528**	0.5104	**0.9101**	**0.9956**	**0.9319**	**0.9790**	**0.9856**	0.6551	0.3923	−0.731	0.6487	0.8569	**0.9602**	0.2202	0.0232	**0.9377**	−0.0917	0.5705	0.6303	0.3792	**0.9503**	0.8026
	3		0.2956	**0.9614**	0.9337		0.5140	0.2345	**0.9542**		0.7155	0.8410	0.8810		0.1021	0.6320	**0.9242**		0.5866	−0.2107	0.5505		0.0783	**0.9698**	**0.9365**
	5			**0.9876**	**0.9226**			**0.9221**	0.7904			0.7932	**0.9947**			**0.9992**	**0.9777**			−0.7183	0.4463			**0.9979**	**0.9096**
	7				0.8691				**0.9617**				**0.9998**				0.7550				−0.1262				0.7391
RA	1	**0.9142**	0.5058	**0.9332**	0.4048	**0.9843**	**0.9999**	**0.9090**	**0.9479**	**0.9968**	0.7333	0.3374	−0.8068	0.8168	**0.9078**	**0.9421**	0.1029	−0.2248	0.8944	−0.1502	0.6639	0.8028	0.4774	**0.9303**	0.7263
	3		0.3976	**0.9435**	0.8846		0.6041	0.1768	**0.9120**		0.7872	0.8077	**0.9309**		0.2096	0.5853	0.8724		0.4951	−0.2679	0.6457		0.1862	**0.9537**	0.8883
	5			**0.9767**	0.8704			0.8977	0.8575			0.7559	**0.9754**			**0.9998**	**0.9957**			−0.7581	0.5493			**0.9999**	0.8538
	7				0.8043				**0.9224**				**0.9904**				0.6719				−0.0076				0.6539
SAE	1	−0.6799	−0.8209	0.4484	0.7826	−0.8346	0.0922	0.3918	0.0689	−0.9474	−0.6211	−0.3839	0.7754	−0.5226	−0.3478	0.4712	**0.9364**	−0.1783	0.5152	−0.7765	−0.8912	−0.5021	−0.8391	0.4414	0.4818
	3		−0.8840	0.4748	0.2278		−0.7477	−0.5316	0.1666		−0.5539	0.2006	−0.589		−0.9586	−0.1128	0.2527		**0.9046**	−0.8465	−0.9019		−0.9652	0.5033	0.2201
	5			0.5780	0.2567			0.3676	−0.7143			0.1186	−0.0332			0.7270	−0.3415			−0.9996	−0.9473			0.7432	0.2880
	7				0.3719				0.1408				−0.1160				0.5470				−0.9656				0.5668
SAF I	1	0.2843	0.8608	0.8761	−0.072	0.0484	0.8672	**0.9045**	0.6876	−0.2076	**0.9711**	**0.9373**	−0.9897	0.4671	**0.9973**	0.8635	−0.3759	−0.9374	0.5651	0.6592	**0.9372**	0.4881	0.8438	0.8799	0.3186
	3		0.7928	0.8614	0.5624		**0.9149**	0.8666	0.6129		**0.9875**	**0.9713**	**0.9935**		0.6576	**0.9972**	0.5410		0.0115	0.5641	**0.9285**		0.6394	0.8444	0.5688
	5			0.7935	0.5375			**0.9154**	**0.9987**			**0.9877**	0.7580			0.6585	**0.9228**			0.0103	0.8772			0.6404	0.5097
	7				0.4314				0.6333				0.8095				0.2457				0.4625				0.2225
SAF II	1	−0.8292	−0.6768	0.8963	0.3962	−0.6726	0.3068	0.8669	**0.9449**	−0.4620	−0.4355	0.2498	−0.8123	−0.9228	−0.1352	**0.9074**	0.0936	**0.9449**	0.6895	−0.2402	0.6709	−0.9318	−0.7004	0.8928	0.7197
	3		−0.7610	**0.9091**	0.8802		−0.5851	0.0859	**0.9081**		−0.3593	0.7502	**0.9343**		−0.8736	0.5085	0.8678		**0.9757**	−0.3551	0.6528		−0.8850	**0.9222**	0.8839
	5			**0.9529**	0.8657			0.8536	0.8623			0.6928	**0.9733**			**0.9939**	**0.9965**			−0.8146	0.5571			**0.9963**	0.8489
	7				0.7987				**0.9188**				**0.9890**				0.6649				0.0017				0.6469
PLS	1	**0.9857**	**0.9680**	**0.9526**	0.8997	**0.9975**	0.6956	**0.9317**	**0.9294**	**0.9467**	**0.9992**	0.3917	−0.2342	**0.9334**	**0.9380**	**0.9600**	0.7211	−0.4571	0.3148	−0.0923	0.0237	**0.9246**	**0.9593**	**0.9501**	**0.9985**
	3		**0.9305**	**0.9612**	**0.9765**		**0.9909**	0.2339	**0.9611**		**0.9925**	0.8407	0.4746		0.8404	0.6315	**0.9817**		−0.2656	−0.2113	−0.0005		0.8272	**0.9697**	**0.9748**
	5			**0.9875**	**0.9825**			**0.9219**	0.3222			0.7928	0.8869			**0.9992**	0.7001			−0.7188	−0.1206			**0.9980**	**0.9880**
	7				**0.9978**				**0.9536**				0.8455				**0.9913**				−0.6518				**0.9879**
CA	1	**0.9264**	0.4431	**0.9997**	0.8452	**0.9893**	**0.9984**	**0.9962**	**0.9648**	**0.9938**	0.6830	0.6339	−0.3414	0.8344	0.8757	**1**	0.6391	−0.2551	**0.9240**	0.1905	0.1354	0.8210	0.4137	**0.9995**	**0.9860**
	3		0.3313	**0.9999**	**0.9945**		0.5459	0.4970	**0.9860**		0.7413	**0.9588**	0.5701		0.1394	0.8236	**0.9968**		0.5557	0.0711	0.1114		0.1158	**0.9993**	**0.9936**
	5			**0.9921**	**0.9972**			**0.9935**	0.4261			**0.9319**	**0.9330**			**0.9480**	0.7756			−0.4951	−0.0088			**0.9403**	**0.9991**
	7				**0.9989**				**0.9813**				0.8999				**0.9704**				−0.5629				**0.9644**

TPC—total phenolic content; CA—caffeic acid; PLS—prolithospermic acid; SAE—salvianolic acid E; RA—rosmarinic acid; SAF I and II—salvianolic acid F isomers I and II. *TAT*: tyrosine aminotransferase, *HPPR*: 4-hydroxyphenylpyruvate reductase, *PAL*: phenylalanine ammonia-lyase, *C4H*: cinnamic acid 4-hydroxylase, *4CL*: 4-coumarate-CoA ligase, *RAS*: rosmarinic acid synthase.

**Table 2:** Primers used for quantitative real-time PCR analysis.

Gene Name	Primers	References	PCR Product Length (bp)
*TAT*	CGCCGACTACCATCACCATTAAGG GCAGAGCCTCCACAACACCTTC	[14]	151
*HPPR*	GACTCCAGAAACAACCCACATT CCCAGACGACCCTCCACAAGA	[43]	138
*PAL*	GGCGGCGATTGAGAGCAGGA ATCAGCAGATAGGAAGAGGAGCACC	[40]	564
*C4H*	CCAGGAGTCCAAATAACAGAGCC GAGCCACCAAGCGTTCACCAA	[43]	186
*4CL*	CGCCAAATACGACCTTTCCTC TGCTTCAGTCATCCCATACCCA	[43]	133
*RAS*	CGCCCTAGTTGAGTTCTACCCTTACGC TCGGATAGGTGGTGCTCGTTTGC	[40]	282
*β-ACTIN*	AGGAACCACCGATCCAGACA GGTGCCCTGAGGTCCTGTT	[14]	267

## Data Availability

The data are contained within the article.
